# Evaluation of the Mechanical, Thermal and Rheological Properties of Hop, Hemp and Wood Fiber Plastic Composites

**DOI:** 10.3390/ma16114187

**Published:** 2023-06-05

**Authors:** Sierra Talcott, Benjamin Uptmor, Armando G. McDonald

**Affiliations:** Department of Forest, Rangeland and Fire Sciences, University of Idaho, Moscow, ID 83843, USA; sierratalcott@gmail.com (S.T.); benuptmor@hotmail.com (B.U.)

**Keywords:** hemp fiber, hop fiber, mechanical properties, rheology, wood–plastic composites

## Abstract

The aim of this study was to evaluate the use of waste natural fibers from milled hop bines and hemp stalks, without chemical treatment, and compare them to a commercial wood fiber for use in wood–plastic composite (WPC) materials. The fibers were characterized (density, fiber size and chemical composition). WPCs were produced by the extrusion of a blend of fibers (50%), high-density polyethylene (HDPE) and coupling agent (2%). The WPCs were characterized for their mechanical, rheological, thermal, viscoelastic and water resistance properties. Pine fiber was about half the size of hemp and hop fibers and thus had a higher surface area. The pine WPC melts had a higher viscosity than the other two WPCs. Additionally, the tensile and flexural strengths of the pine WPC were higher than those of hop and hemp WPCs. The pine WPC was also shown to have the least water absorption followed by hop and hemp WPCs. This study highlights that different lignocellulosic fibers influence their WPC properties. The properties of the hop- and hemp-based WPCs were comparable to commercial WPCs and can be improved by further milling/screening the fibers to a smaller particle size (volumetric mean of ~88 μm) to increase their surface area, fiber–matrix interactions and improve stress-transfer.

## 1. Introduction

The wood–plastic composites (WPCs) market size is USD 5.6 billion globally in 2021 with about a 10% compounded growth [1]. WPCs have relied on a steady stream of sawmill and secondary manufacturing residues for wood fiber [2]. In geographical locations where wood residues are not available, alternative natural fibers can be used in WPC applications [3]. There are various natural fibers available regionally, such as wheat straw [4], rice husks [5], bamboo [6], rattan canes [7], corn cobs [8] and banana stalks [9] that could be used as alternatives to wood fiber [10]. In the USA there are new fiber sources available, notably from hemp and hops, that could be used as a wood replacement.

Hemp is a growing industry in the U.S. based primarily on cannabidiol (CBD) which in 2017 hit USD 291 million and is expected to reach USD 1.65 billion by 2023 [11]. Since there has been an oversupply of CBD, which contributes to CBD price volatility, coproducts from hemp are required (such as seed oil and fiber) to stabilize the economics. Hemp stalks’ (fiber) mass can be quite significant and is now considered a low or no value product [11]. Regionally, most of the stalks from CBD production are currently burnt or disposed of. Hemp stalks are processed by mechanical decortication followed by carding to produce long bast fibers and short hurd fibers [12]. The long bast hemp fibers have good properties such as tensile strength (550–900 MPa) and stiffness (40–65 GPa) and can be used in clothing [13]. By utilizing the inherent properties (e.g., stiffness, fiber length and aspect ratio) of hemp bast fibers [12,13], which can be grown and processed locally, is an ideal reinforcing fiber for use in composite materials. The short hurd fibers, with less desirable fiber properties, can also be used in thermoplastic composite materials.

Hop bines are stalks that climb around a rope support, are cut back annually after harvesting the hop flower for beer production and are disposed of by burning or composting. Hop bines have not been realized and utilized for their fiber. However, a recent study had produced a hop fiber pulp by chemical treatments (2% NaOH followed by 1% NaClO) and used the pulp in a mixed plastic–hop fiber composite [14]. Another study extracted hop fibers by chemical treatment (NaOH and acetic acid) and used them in polypropylene composites [15]. The hop fiber composites had relatively low strength and modulus but had an elongation higher than that of hemp-based composites [16]. These studies clearly show the potential of using hop bine waste for producing fibers by a chemical puling process. Using mechanically refined, non-chemically pulped, hop fibers is a more sustainable option for producing fibers.

The aim of this study was to evaluate the use of waste natural fibers from hop bines and hemp stalks, without chemical treatment, just milling, and compare it to a commercial wood fiber for use in WPC materials. The natural and wood fibers were characterized by particle size, density and chemical composition. The fibers were compounded in combination with high density polyethylene (HDPE) and coupling agents to form extruded composite materials. The rheological, thermal, physical and mechanical properties of the various composite materials were determined and compared.

## 2. Materials and Methods

### 2.1. Materials

**Figure 1:**
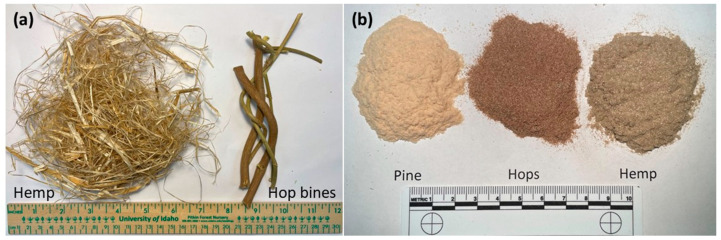
(**a**) Photograph of hemp fiber and hurd and hop bines and (**b**) milled pine, hop and hemp fibers.

Hop bines (*Humulus lupulus*) (Figure 1a) were provided by Maggie Zee and sourced from Yakima Chief Ranches (Yakima, WA, USA). The hemp hurd and fiber mixture (Figure 1) was provided by Shayne Kimball (Kimball Farms, Joseph, OR, USA). The dried hop bines and hemp fibers were Wiley milled to pass through a 0.5 mm screen and dried in an oven at 105 °C for 4 h prior to use (Figure 1b). Pine fibers (10020, American Wood Fibers, Schofield, WI, USA) were dried (105 °C for 4 h) prior to use (Figure 1b). High-density polyethylene (HDPE, Equistar Petrothene LB 0100-00, MFI = 0.3 g/10 min, and density = 0.950 g/cm^3^) and maleated polyethylene (MAPE, Polybond 3029, Crompton Corp., Middlebury, CT, USA) coupling agents were used as received. 

### 2.2. Fiber Characterization

The particle size distribution of the fibers was determined in the dry state using a LS13 320 XR Beckman Coulter particle size analyzer equipped with a Tornado Dry Powder System (Indianapolis, IN, USA). Brightfield optical microscopy (Olympus BX53, 20× objective and equipped with a DP28 camera; Waltham, MA, USA) was employed to determine the aspect ratio of 50 fibers by measuring the length and width using the Olympus CellSens v4.1 software (Waltham, MA, USA). Analyses were performed in triplicate. The density of fibers (1.0 g) was determined by gas pycnometry on an Ultra-Pycnometer 1000 (Quantachrome, Boynton Beach, FL, USA) with N_2_.

Fiber samples (4–5 g) were Soxhlet extracted with CH_2_Cl_2_ (150 mL) for 16 h according to ASTM D1108. The CH_2_Cl_2_ extract was concentrated to dryness to determine extractives yield gravimetrically. The lignin content (Klason plus acid soluble) was determined on the extractive’s free fiber according to a modified ASTM D1106. Specifically, the extractive free fiber (200 mg) was digested in 72% H_2_SO_4_ (2 mL) for 1 h at 30 °C then diluted into 4% H_2_SO_4_ and subjected to secondary hydrolysis in a pressure cooker (116 kPa and 120 °C) for 30 min. The hydrolyzate was filtered to obtain Klason lignin content gravimetrically and the acid soluble lignin determined colormetrically at 205 nm (Genesys 50, Thermo Scientific, Madison, WI, USA) using an extinction coefficient (ε) of 110 L/g·cm [17]. All analyses were conducted in duplicate. Neutral carbohydrates analysis was carried out according to ASTM E 1758 on the secondary hydrolysates by HPLC with inositol added as an internal standard [18]. Separation was achieved using two Rezex RPM columns (7.8 mm × 300 mm, Phenomenex, Torrance, CA, USA) in series at 90 °C on elution with water (0.5 mL/min) with differential refractive index detection (Waters model 2414, Milford, MA, USA).

### 2.3. Composite Preparation

The dried fiber (250 g), HDPE powder (240 g) and MAPE powder (10 g) were blended in a Kitchen aid for 10 min to obtain a homogeneous mixture. The blended mixture (500 g batch) was fed using a weight loss feeder (K-Tron, Sewell, NJ, USA) at 0.5 kg/h into an 18 mm diameter and length/diameter of 40 co-rotating twin screw extruder with two kneading zones (Leistritz, Allendale, NJ, USA). The extruder was operating at 200 rpm with barrel and die temperature zones of 160 °C, and the mixture was extruded into a ribbon (3.3 mm × 50 mm) [14].

### 2.4. Rheological Measurements

Viscosity measurements were determined at 190 °C using a capillary rheometer (Instron Model 3213, Norwood, MA, USA) connected to an Instron 5500R-1137 universal testing machine (44 kN load cell) [5]. Cross head speeds of 0.6, 2, 6, 20, 60 and 100 mm/min were used with a barrel diameter of 9.5504 mm, and data were acquired using the BlueHill v3.3 software. The dies were 14 and 27 mm long with a diameter of 1.4 mm and an entrance angle of 70°. Samples (8 g) were loaded in the barrel and equilibrated at 190 °C for 10 min prior to testing. Each sample was run in triplicate. Since the L/D ratio was <200, a Bagley correction was used to correct for the effect of the drop in pressure during measurement [5].

### 2.5. Thermal Analysis

Differential scanning calorimetery (DSC) was performed on a Perkin Elmer DSC-7 (Shelton, CT, USA) instrument from 25 to 200 °C at 10 °C/min under N_2_ (20 mL/min). The DSC was calibrated with indium. HDPE crystallinity was calculated using an HDPE fusion enthalpy of 293 kJ/g [19]. Thermogravimetric analysis (TGA) was performed on a Perkin Elmer TGA-7 (Shelton, CT, USA) instrument from 30 to 900 °C at 20 °C/min under N_2_ (30 mL/min). The TGA was calibrated with alumel, perkalloy, nickel and iron standards. Dynamic mechanical analysis (DMA) was carried out in 3-point bending mode (15 mm span) on rectangular bars (4 mm × 2 mm × 20 mm) with a Perkin Elmer DMA-7 (Shelton, CT, USA) instrument from −50 to 120 °C at a heating rate of 3 °C/min, 0.05% strain, and 1 Hz. Data were analyzed using the Pyris v13.3 software.

### 2.6. Mechanical Properties

Tensile dog-bone specimens (ASTM D638 type I) were prepared by drum sanding (Grizzly model G0716) the extruded ribbon to obtain a flat surface and then routering (Dremmel plunger router with a custom-built jig) into dog-bone specimens. The composites and HDPE samples (7 replicates) were tensile tested on an Instron 5500R-1132 universal testing machine (Norwood, MA, USA) equipped with a 5 kN load cell and model 3542 extensometer (Epsilon Technology Corp, Jackson, WY, USA) with a cross head speed of 5 mm/min according to the ASTM D638. Three-point flexural tests (strength and modulus) were performed on machined, extruded specimens (3.1 × 13 × 110 mm^3^, 6 replicates) according to ASTM Standard D 790 with a crosshead speed of 1.3 mm/min and a span of 50 mm until specimen failure or 5% strain, whichever occurred first on an Instron 5500R-1132 universal test machine. Data were collected and processed using Bluehill v3.3 software (Instron). The specimens were conditioned for at least 7 days at 60–65% relative humidity and 21 °C prior to testing.

### 2.7. Water Soak Test

Weight gain of WPC samples (25 mm Ø × 3 mm), in quadruplicate, were submerged in a water bath, and gravimetric measurements were taken every week for 5 weeks at room temperature.

## 3. Results and Discussion

### 3.1. Fiber Characterization

**Figure 2:**
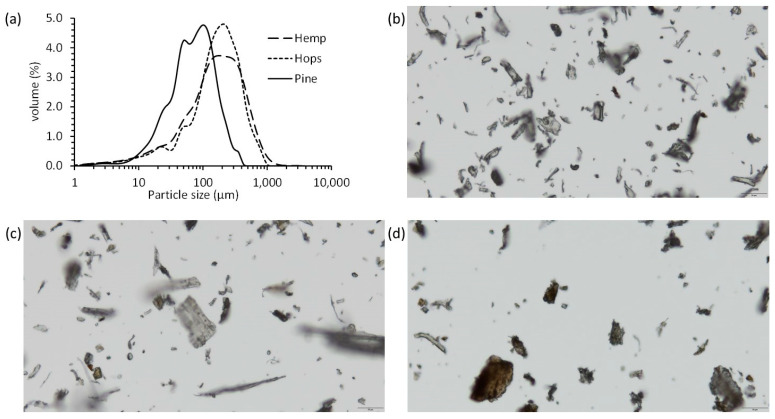
(**a**) Particle size distribution of pine, hemp and hop fibers and light micrographs of (**b**) pine fiber, (**c**) hemp fiber, and (**d**) hop fiber. Scale bar = 50 µm.

The hemp hurd/fibers and hop bines were milled and screened to <0.5 mm to produce a uniform fiber for use in composite materials (Figure 1b). This process did not use chemicals to produce the fine fiber as in other studies [14]. Natural fibers (bamboo, rice husks, and corn cob) produced using the 0.5 mm sized screen have been shown to produce fibers suitable for use in thermoplastic composites, while large screen sized fibers (<1 mm or <2 mm) produced composites with reduced properties [5,6,8]. The fiber size distribution for pine, hemp and hop fibers was determined by light scattering (Figure 2a). Light scattering measurements assume a spherical particle shape to determine diameter. Mean, median and mode are statistical definitions for the average, the middle value of a population and the value that most frequently appears in a population, respectively. Commercial pine fiber, commonly used in WPCs [20,21], had the shortest fiber with volumetric mean, median and mode diameter values of 88.3, 71.8 and 103 µm, respectively. Hemp fiber was largest with mean, median and mode particle size values of 222, 164 and 185 µm, respectively, while hop fibers were 209, 173 and 204 µm, respectively. The calculated specific surface area, based on a spherical geometry, for pine, hemp and hop fibers were, respectively, 1432, 1222 and 1056 cm^2^/mL. Stark and Rowlands observed that wood fiber aspect ratio influenced WPC strength [22]. Therefore, the fibers were also analyzed for shape by optical microscopy and showed a range of fine and large fibers (Figure 2b–d). The range in length for the pine, hemp and hop fibers were 3.3–59 µm, 3.6–176 µm and 4.1–101 µm, respectively. The calculated aspect ratios (length/width) for pine, hemp and hop fibers were 2.9 ± 1.6, 3.7 ± 3.5 and 2.5 ± 1.5, respectively. Englund had observed an aspect ratio of 3–6 for various hardwood fibers [23], while Stark and Rowland observed an aspect ratio of 3.3–4.5 for screened commercial pine fibers [22].

The fibers were also analyzed for density and chemical composition (ash, extractives, lignin and carbohydrate) and the results are given in Table 1. Pine fiber was shown to have the lowest density at 1.46 g/cm^3^ while hop fiber was the highest at 1.53 g/cm^3^. These cell wall density values are consistent with the literature for wood and natural fibers (approximately 1.5 g/cm^3^) [8,24].

Ash can contribute to abrasion of the process equipment during extrusion or molding and its content is important. The ash content ranged from 0.37% for pine to 4.59% for hemp fiber, which is in the range for wood and natural fibers [25]. Extractives (lipids) are known to act as lubricants in processing plastic and WPCs and can negatively influence flexural properties [26]. The CH_2_Cl_2_ extractives content for the hop and hemp fibers were low (1.1 and 1.5%, respectively) while the pine fiber had a significantly higher extractives content of 7.5%. Hemp fiber lipid content has been reported to be between 0.2 and 0.7% [27]. Acetone extractives content of commercial pine fiber has been reported at 3.2% [28], which is 57% lower than the CH_2_Cl_2_ extractives in this study, while Saputra et al. obtained an extractives content of 5.4% for pine [26]. CH_2_Cl_2_ extractives content of ponderosa pine has been reported at 5.1% [29]. This high extractives content in pine fiber is likely to improve the processability of the composite.

The lignin content of the fibers ranged between 16% and 28%. The lignin content for pine (28%) was comparable with literature values (25–33%) [29,30]. Hemp fiber had a lignin content of 26.7% which was significantly higher than that reported by Zimniewska [27] at 2–10%. This discrepancy may be due to the fact that the hemp fibers were not chemically processed and had a higher lignin content than chemically treated, retted and isolated fibers. Hops had a higher lignin content (15.9%) than reported by Reddy (6%) for chemically extracted fibers [16].

Carbohydrate analysis of the fibers showed that they were mainly composed of cellulose followed by hemicellulose (xylan, mannan and galactan) (Table 1). Cellulose contributes to fiber tensile strength and therefore is an important variable for composite properties [30]. Cellulose content for pine, hemp and hops was 42, 40 and 51%, respectively. Pine cellulose content was in line with reported softwood values (37–43%) [30]. Literature values for hemp fibers were higher and ranged between 52 and 78% [27]. Reddy reported a higher cellulose content of 84% for chemically extracted hop fiber [16].

### 3.2. Composites Characterization

#### 3.2.1. Capillary Rheology

**Figure 3:**
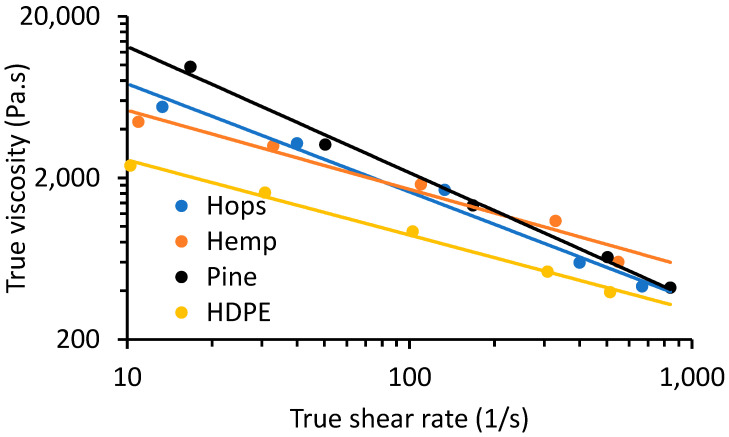
Flow curves of true shear rate ($\dot{\gamma }$) against true viscosity (η) for HDPE and pine, hemp and hop WPCs at 190 °C.

The melt flow characteristics of the composites and HDPE was carried out by capillary rheometry according to ASTM D3835, and the Bagley and Weissenberg–Rabinowitsch corrections were applied to the results obtained by using two die lengths to obtain true shear rate ($\dot{\gamma }$ and true viscosity (η) values (Figure 3) [5,31]. The rheological data obtained shows a general trend of η decreasing with $\dot{\gamma }$, which is characteristic of shear thinning behavior of a non-Newtonian fluid [31,32]. A reduction in η at high $\dot{\gamma }$ was due to disentanglement and less molecular interactions among polymer chains [33]. To quantitatively assess the shear thinning behavior, the rheological data (η and $\dot{\gamma }$) were fitted to the power law model (Equation (1)):

(1) $$\eta \;(\dot{\gamma })=K{\left(\dot{\gamma }\right)}^{n-1}$$

where, K is the consistency coefficient and *n* is the non-Newtonian or flow behavior index (Table 2) [34]. Since the parameter *n* for the samples ranged between 0.219 and 0.535 this is consistent with a pseudo-plastic material. The goodness of fit for the models, R^2^, was ≥ 0.97. The η of the pine, hemp and hop WPC plus HDPE were compared at $\dot{\gamma }$ of 100 s^−^^1^ (Table 2). HDPE had an η of 884 Pa·s and the η increased with the addition of fiber. Pine WPC had the highest η at 2155 Pa·s followed by hemp (1697 Pa·s) and hop (1636 Pa·s). For comparison, Orji and coworker observed an η of 1621 Pa·s but at a shear rate of 30 s^−^^1^ and 190 °C for recycled HDPE, and the η increased with the addition of 50% rice hulls (<0.5 mm particle size) to 3548 Pa·s [5]. Ewurum et al., while studying comingled mixed plastic waste (mainly containing polyethylene-terephthlate, PE, polyethylene-co-vinyl acetate and paper), obtained an η of 1487 Pa·s, and by adding 10% pulped hop fiber the η increased by 32% [14]. These results clearly show how different lignocellulosic fiber species greatly influence the rheological properties of WPCs, and this has been observed by Li and Wolcott comparing commercial maple versus pine fibers [35]. The observations of increased viscosity with a decrease in fiber particle size are consistent with findings by Mazzanti and Mollica [31]. Hristov and coworkers observed that as particle size decreased, wall slip lowered as a result of a thinner slip layer [36].

#### 3.2.2. Thermal Analysis

**Figure 4:**
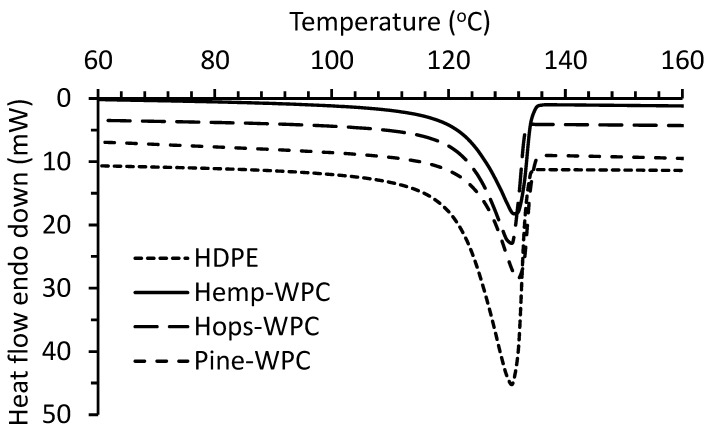
DSC thermograms of HDPE and fiber WPCs.

The thermal properties (melt onset temperature (T_onset_), T_m_ and X_c_) of the composites and HDPE were determined by DSC (Figure 4) and the results are given in Table 3. The T_onset_ for HDPE was 121 °C and slightly increased with the addition of fiber (122–124 °C). The T_m_ for all samples was close; around 131–132 °C. A similar T_m_ for pure HDPE at 131 °C was observed by Wang et al. [37]. The X_c_ for HDPE was 61.5%, which slightly increased by 0.6–1.1% with the addition of hemp and hop fiber and increased by 2.9% with pine fiber.

**Figure 5:**
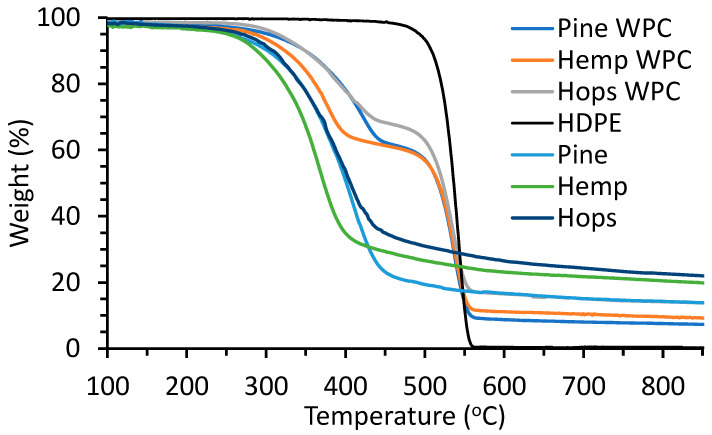
TGA thermograms of fiber, HDPE and fiber WPCs.

The thermal degradation behavior of the fiber, HDPE, and composites was determined by TGA as shown in Figure 5. The thermal degradation transition temperatures (T_95_ and T_onset_) and residue at 850 °C are given in Table 4. Total degradation for HDPE occurred in one step with a T_onset_ of 518 °C for HDPE due to radical chain mechanics and rapid chain scission [38]. Awad et al. observed a lower thermal breakdown of HDPE at 462 °C, and this could be due to a lower heating rate of 10 °C/min used [39]. The hemp fiber had the lowest T_onset_ of 314 °C followed by hop (323 °C) and pine (335 °C). The fibers degraded over a 250 to 450 °C range, and this is common for lignocellulosic fibers [40]. The composites samples showed two onset temperatures, the first (T_onset1_) associated with fiber degradation (324–354 °C) and the second (T_onset2_) associated with HDPE decomposition (512–514 °C). These composite samples behaved like WPCs made with different wood species and modified wood fibers [41,42]. The hemp WPC behaved similar to those of Xanthopoulou et al. for hemp fiber–HDPE composites [43]. The residual mass (at 850 °C) for the fibers and WPCs is a combination of ash (minerals) and char (fixed carbon) under a nitrogen environment. The residual mass for HDPE was minimal. The ash contents were low for the fibers (0.4–4.6%) while the residual mass was 13.8–21.3%, suggesting that this was mainly char.

**Figure 6:**
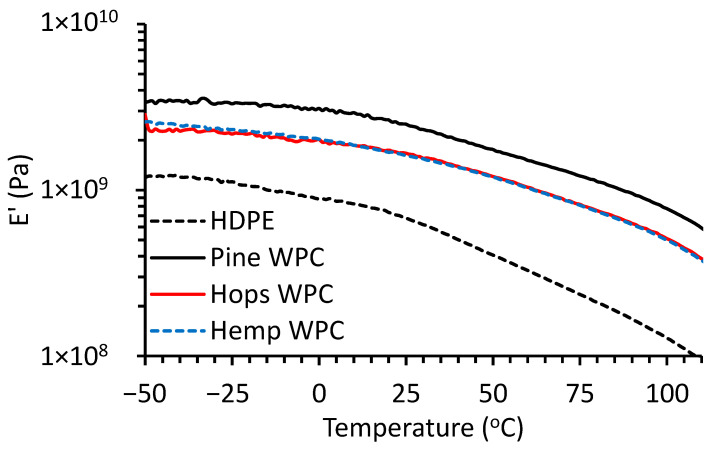
Dynamic mechanical analysis thermogram of storage modulus (E’) of HDPE and fiber composites.

The viscoelastic properties, storage modulus (E′), of the composites and HDPE were determined by DMA. The values at 30 °C are given in Table 5 and the thermograms are shown in Figure 6. The pine WPCs had the highest E′ (at 30 °C) value at 2.32 GPa while HDPE had the lowest at 0.62 GPa. The hemp and hop composites had an E′ of about 1.55 GPa. The high E′ for pine could be attributable to having smaller particles than hop and hemp fiber thus having a larger surface area for interaction with the matrix and better stress transfer [21,22,44].

#### 3.2.3. Mechanical Properties

**Figure 7:**
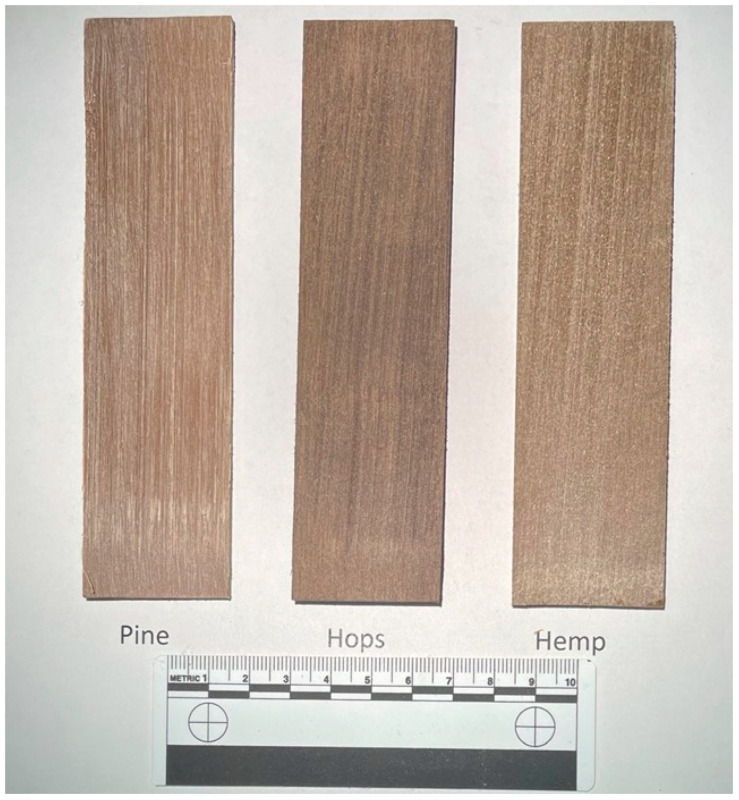
Photograph of the pine-, hemp- and hop-extruded WPCs.

**Figure 8:**
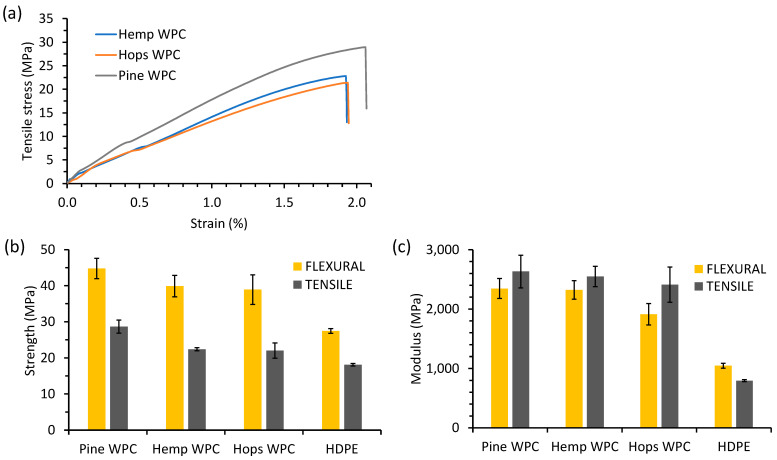
(**a**) Tensile stress versus strain curves, (**b**) tensile and flexural strength data and (**c**) Young’s and flexural moduli data of the hop, hemp and pine fiber composites and HDPE.

The extruded composite ribbons were sanded prior to testing (Figure 7). The tensile stress versus strain curves of selected fiber WPCs is shown in Figure 8a. The flexural and tensile properties of the fiber composites and HDPE are shown in Figure 8b,c. The tensile strength for HDPE was 18.1 MPa and increased upon addition of hop, hemp and pine fiber to 22.0, 22.4 and 28.7 MPa, respectively. The higher pine WPC tensile strength values could be attributed to having smaller fibers and larger surface area/volume than the hop and hemp fibers thus having better stress transfer [21,44]. Tensile strength values were higher than those reported by Singh et al. for 30% hemp–HDPE composites (17.4 MPa) [45] and Xanthopoulou et al. [43] for 50% hemp–HDPE composites (14 MPa) and lower than that obtained by Luo et al. for a 40% hemp–HDPE composite (46 MPa) [46]. Zou et al. obtained a low tensile strength value of 6.8 MPa for compression molded hop fiber–polypropylene composites [15]. The tensile (Young’s) modulus for the fiber composites for pine was 2.63 GPa, followed by hemp (2.55 GPa) and hop (2.41 GPa), but these were not significantly different. Luo et al. obtained Young’s modulus values of 1.3–2.3 GPa for 40% hemp fiber–HDPE composites [46], Xanthopoulou et al. [43] obtained 1.2 GPa for 50% hemp–HDPE composites, while Zou et al. obtained a value of 0.4–0.7 GPa for hop fiber–polypropylene composites [15].

The flexural strength for the hop, hemp and pine fiber composites and HDPE were, respectively, 38.9, 39.8, 44.8 and 27.4 MPa (Figure 8b,c). The flexural strength values for these composites were higher than WPCs made from various wood species with HDPE (21–25 MPa) [41], molded hop fiber–polypropylene composites (6–13 MPa) [15], and a 30% hemp–HDPE composite (17.4 MPa) [44]. The flexural strength of these composites was at least 50% higher than commercial PE-based WPCs [47]. The high flexural strength of the pine WPC as compared to hop and hemp WPCs could be due to having smaller fibers and better interaction with the HDPE matrix [44]. The flexural modulus for the pine and hemp fiber composites was 2.34 GPa (not significant) and was significantly different from the hop composite (1.91 GPa) and the HDPE (1.05 GPa). The flexural moduli were comparable (1.5–2.6 GPa) to those of Fabiyi et al. for HDPE-based WPCs [41] but were higher than hop fiber–polypropylene composites (1.6 GPa) [15]. The flexural moduli of the pine and hemp WPCs were at least 15% lower than commercial WPCs at 2.7 GPa [47]. These results clearly show that these plant fibers are suitable for WPC applications.

#### 3.2.4. Water Soak Tests

**Figure 9:**
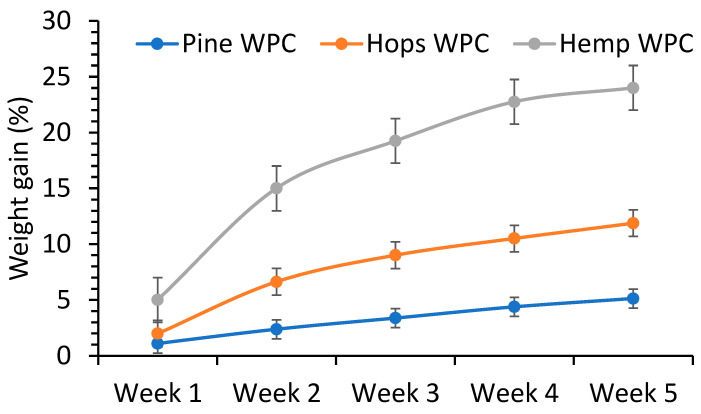
Plot of weight gain against time for hop, hemp and pine fiber composites in a water soak test.

The water soak (weight gain) properties of the composites were measured over 5 w and show how these materials behave in exterior use (Figure 9). The water weight gains increased with soaking time but did not reach a plateau after 5 w. Pine composites showed the least weight gain at 5.1% at 5 w, followed by the hop (12%) and hemp composites (24%). An explanation for why the pine WPC had the least weight gain could be attributed to its high extractives content, making the fibers more hydrophobic than hemp and hop fibers. The results obtained were within the range for WPCs made from different wood species, between 8% (black locust) and 22% (ponderosa pine), at about week 5 soaking time [48]. Englund had shown a weight gain of 10–15% at 5 weeks for a polypropylene-based WPC made with various hardwood species [23]. Rice-hull’s HDPE composites were shown to have a low weight gain of around 3–4% at week 5 which increased to 8–10% at week 18 [5]. The three composites were shown to have lower weight gain (0.2–0.7%) compared to a commercial WPC at 1.2% after 24 h [47]. These results clearly show that these fibers are suitable for use in WPC applications.

## 4. Conclusions

Milled hop and hemp fibers were compared to commercial pine fiber for their suitability for use in WPCs. The composition of the three fibers (extractives, lignin and carbohydrate contents) was shown to be different based on plant species. The hemp and hop fibers were about two times larger in size than the pine fiber, which resulted in the pine fiber having a higher calculated specific surface area. All three fibers were shown to have an oblong shape with an average aspect ratio of 2.5–3.7, which will help as reinforcing material.

WPCs were successfully prepared with three types of fibers as reinforcing filler by an extrusion process. The pine WPC melts had a higher viscosity than the other two composites, indicating different melt processability. The tensile and flexural strengths of pine WPC were higher than those of hop and hemp WPCs. This is likely associated with improved enhanced interfacial interaction, with pine having a higher specific surface area. Generally, the tensile and flexural moduli for the WPCs were comparable. The hop and pine fibers and WPCs had more thermal stability relative to the hemp fibers and WPC. In water soak tests, pine WPC had the lowest water absorption followed by hop and hemp WPCs. This study highlights that different types of fiber influence their WPC properties. To improve the performance of hop- and hemp-based WPC, it is believed that milling the fibers to a smaller particle size would increase their specific surface area, thus improving interactions between the fiber and the plastic matrix and their WPC mechanical and physical properties, comparable to pine WPC. The hemp and hop fiber-based WPCs were shown to perform as well, or better, than a commercial WPC decking product and can be a direct replacement fiber in this application. This will provide an outlet for farmers to sell these fibers as a coproduct and improve their economic viability.

## Figures and Tables

**Table 1:** Composition of wood, hemp and hop fiber.

Components	Pine	Hemp	Hop
Density (g/cm^3^)	1.455 ± 0.003	1.518 ± 0.001	1.526 ± 0.001
Ash (%)	0.37 ± 0.01	4.59 ± 0.04	3.14 ± 0.02
Extractives (%)	7.53 ± 0.20	1.51 ± 0.10	1.12 ± 0.04
Klason lignin (%)	27.5 ± 0.6	25.2 ± 0.6	13.4 ± 0.2
Acid soluble lignin (%)	0.55 ± 0.00	1.48 ± 0.03	2.47 ± 0.01
Total lignin (%)	28.1	26.7	15.9
Glucan/cellulose (%)	42.0 ± 0.00	39.5 ± 0.3	50.5 ± 0.5
Xylan (%)	8.0 ± 0.1	11.7 ± 0.1	10.1 ± 0.1
Galactan (%)	4.6 ± 0.1	3.4 ± 0.1	5.2 ± 0.1
Arabinan (%)	0.9 ± 0.05	0.8 ± 0.05	0.4 ± 0.05
Mannan (%)	10.9 ± 0.1	1.4 ± 0.1	3.2 ± 0.1
Total carbohydrates (%)	66.5	56.8	69.4

**Table 2:** Power law fit equation of rheological data for HDPE and pine, hemp and hop WPCs formulations.

Formulation	Equation	K (Pa·s)	*n*	R^2^	η at 100 s^−1^ (Pa·s)
HDPE	y = 7523x^−0.465^	7523	0.535	0.990	884
Pine WPC	y = 78,591x^−0.781^	78,591	0.219	0.990	2155
Hemp WPC	y = 16,132x^−0.489^	16,132	0.511	0.974	1697
Hop WPC	y = 35,618x^−0.669^	35,618	0.331	0.980	1636

**Table 3:** Thermal data for HDPE and fiber composites determined by DSC.

Sample	T_onset_ (°C)	T_m_ (°C)	X_c_ (%)
HDPE	121.3	130.9	61.5
Pine WPC	124.1	131.9	64.4
Hemp WPC	122.4	131.4	62.1
Hop WPC	122.3	130.7	62.4

**Table 4:** Thermogravimetric data (T_95_, T_onset_ and residue content) of fibers, HDPE and fiber WPCs.

Sample	T_95_ (°C)	T_onset1_ (°C)	T_onset2_ (°C)	Residue at 850 °C (%)
HDPE	493	518		0.3
Pine fiber	254	335		13.8
Hemp fiber	251	314		19.9
Hop fiber	274	323		21.3
Pine WPC	302	354	514	7.3
Hemp WPC	286	324	515	9.1
Hop WPC	314	326	512	13.8

**Table 5:** Storage modulus (E′) for HDPE and fiber composites determined by DMA at 30 °C.

Sample	E′ at 30 °C (GPa)
HDPE	0.619
Pine WPC	2.32
Hemp WPC	1.54
Hop WPC	1.56

## Data Availability

Data will be provided when requested.
